# Post-transcriptional mapping reveals critical regulators of metastasis

**DOI:** 10.18632/oncoscience.207

**Published:** 2015-08-20

**Authors:** George S. Hussey, Breege V. Howley, Philip H. Howe

**Affiliations:** Department of Biochemistry and Molecular Biology, Hollings Cancer Center, Medical University of South Carolina, Charleston, SC, USA

**Keywords:** TGFβ, translational regulation, hnRNP E1, activin A, metastasis

During TGFβ-induced EMT, the transition to a mesenchymal phenotype requires not only transcriptional regulation, through factors such as Zeb1/2 and Snail, but also coordinated post-transcriptional regulation, via microRNAs and RNA-binding proteins, such as hnRNP E1 [1]. Translational control has been shown to play an important role in numerous pathophysiological processes including inflammation [2], and cancer progression [3], and is hypothesized to be energetically and kinetically efficient thereby allowing for more well-defined and rigorous regulatory checkpoints. Structural elements of the mRNA, including the 5′ cap, 5′-UTR, 3′-UTR, poly(A) tail, and *trans*-acting factors such as RNA binding proteins are important determinants of post-transcriptional control and have been implicated as possible molecular targets for therapeutic intervention [4]. Numerous studies from our laboratory have clearly shown that regulation of gene expression at the post-transcriptional level plays an indispensable role during epithelial-mesenchymal transition (EMT) and metastasis. We have shown previously that hnRNP E1 is a key regulator of TGFβ-induced EMT. Silencing of this protein induces a transition of epithelial cells to a mesenchymal phenotype, resulting in enhanced migration, invasion and tumorigenesis [5, 6].

Recent work from our lab has built upon these findings by demonstrating that the inhibin βA transcript is translationally regulated by hnRNP E1 during TGFβ-induced EMT, and the consequent establishment of Activin A autocrine and paracrine signaling is capable of altering the tumor microenvironment and promoting a permissive niche at both the primary tumor site and at secondary metastatic foci [7]. Based on cell culture studies, upregulation of inhibin βA protein was found to occur within 3 hours of TGFβ treatment with a steady increase in secreted levels of the inhibin βA homodimer, Activin A, detected by 24 hours, without a concomitant increase in transcript expression. These results are demonstrative of the emerging discordance between gene transcription and post-transcriptional control processes. By utilizing polysome profiling, we could direct our analyses exclusively towards the translational compartment, thereby confirming the post-transcriptional activation of inhibin βA following TGFβ treatment. Furthermore, upregulation of inhibin βA coincided with the release of the RNA binding protein hnRNP E1 from the transcript, a finding that is consistent with our previous research which shows loss of interaction of hnRNP E1 with ILEI and Dab2, two transcripts that are similarly translationally regulated by TGFβ [5, 6].

The functional significance of inhibin βA upregulation was demonstrated by enhanced cell migration and invasion of mammary epithelial cells when treated with recombinant Activin A. Furthermore, silencing of inhibin βA attenuated the invasive phenotype *in vivo*. These observations come in marked contrast with the observation that despite its ability to promote migration and invasion, Activin A alone is not capable of inducing a complete mesenchymal transition nor does this ligand appear to enhance the transition induced by TGFβ. This lack of EMT induction may be due to deficient Smad2/3 activation or the requirement of parallel non-canonical pathways activated by TGFβ, but not by Activin A, that are required alongside Smad2/3 signaling to induce an EMT. Inhibin βA can therefore be classified as a factor that promotes the invasive phenotype associated with EMT induction and a recently established member of a cohort of ‘EMT signature’ genes regulated by TGFβ at the translational level [8]. Thus, this TGFβ activated translational mechanism regulates a distinct set of mRNA transcripts that likely work in concert to modulate key cellular pathways contributing to metastatic progression and tumor development. These findings highlight the importance of translational control during cancer progression, and demonstrate the utility of post-transcriptional mapping as a powerful tool for interrogation of disease onset and progression.

Mechanistically, the RNA binding protein hnRNP E1 binds to the 3′-UTR of these mRNAs and regulates their translation in a TGFβ-dependent manner. This represents an unusual case of agonist- or stimulus-dependent upregulation of translation through a 3′-UTR element. Thus, the elucidation of this post-transcriptional regulatory pathway is of note in that it not only identified ‘EMT signature’ genes, but also provided mechanistic information as to how they control TGFβ-mediated EMT. Our data demonstrate that phosphorylation of hnRNP E1 is the trigger for the reversal of translational silencing, resulting in a temporal and spatially controlled increase in protein expression. In the dephosphorylated state hnRNP E1 mediates translational silencing, whereas its phosphorylation, in response to TGFβ, relieves translational silencing and allows transition to the mesenchymal phenotype. These findings may have significant implications towards potential prognostic and clinical applications. If in fact the EMT transition is reflective of the metastatic process, then one might predict that the phosphorylation status of hnRNP E1 may be indicative of metastatic progression and the prognosis of patients.
